# Lower brain‐derived neurotrophic factor levels and hippocampal subfield volumes are associated with high diabetes risk in later life

**DOI:** 10.1002/alz.093248

**Published:** 2025-01-09

**Authors:** Jennifer Hanna Al‐Shaikh, Olivia R Ghosh‐Swaby, Ali R. Khan, Jane Thornton, Timothy J Bussey, Lisa M Saksida, Teresa Liu‐Ambrose, Lindsay S Nagamatsu

**Affiliations:** ^1^ Schulich School of Medicine and Dentistry, London, ON Canada; ^2^ Western University, London, ON Canada; ^3^ Fowler Kennedy Sport Medicine Clinic, Western University, London, ON Canada; ^4^ Robarts Research Institute, London, ON Canada; ^5^ Vancouver Coastal Health Research Institute, Vancouver, BC Canada; ^6^ Centre for Aging SMART, Vancouver Coastal Health Research Institute, Vancouver, BC Canada; ^7^ University of British Columbia, Vancouver, BC Canada; ^8^ Djavad Mowafaghian Centre for Brain Health, Vancouver, BC Canada

## Abstract

**Background:**

Older adults with type 2 diabetes (T2D) are more likely to develop Alzheimer’s disease (AD) due to impaired brain metabolism. Although the underlying mechanisms of this relationship are largely unknown, lower levels of brain‐derived neurotrophic factor (BDNF) –which promotes hippocampal neurogenesis in adulthood– and atrophy of the hippocampus are evident in patients with T2D and dementia, possibly linking the two conditions. The hippocampus is comprised of multiple subfields, each with their respective functions, cellular composition, and age‐related sensitivity. In particular, the dentate gyrus, known for its key role in memory processing, is one of the only regions in the brain capable of producing new neurons through BDNF. At the onset of dementia, the rapid loss of hippocampal tissue results in functional disconnection with other areas of the brain involved in cognition, making hippocampal atrophy a biomarker for cognitive decline. Understanding region‐specific hippocampal atrophy during the early stages of diabetes may provide insight into the link between T2D and brain atrophy that is characteristic of AD. Therefore, our study investigates the relationship between BDNF levels and hippocampal subfield volumes in older adults at risk for T2D.

**Method:**

Sixty older adults (aged 60‐80 years, mean = 68.64, 72.34% female) at risk for diabetes completed demographic and medical history questionnaires. Diabetes risk was assessed using HbA1c, BMI, and a diabetes questionnaire. BDNF serum levels were analyzed from blood samples using an enzyme‐linked immunoassay test (ELISA). Anatomical T1 and T2 weighted brain images were collected via 3T MRI and analyzed using HippUnfold – a novel segmentation tool that accounts for individual variability in the folding patterns of hippocampal subregions.

**Result:**

Preliminary results show that higher diabetes risk was associated with lower BDNF levels and reduced hippocampal subfield volumes. In addition, BDNF serum levels were predictive of hippocampal subfield volumes, specifically in the dentate gyrus.

**Conclusion:**

Findings demonstrate a relationship between diabetes risk factors, BDNF, and hippocampal subfield volumes (i.e., dentate gyrus) in regions where neurogenesis continues throughout adulthood. As life expectancy and diabetes incidence increases, our study urges the development and application of interventions aimed at preventing the progression of diabetes‐related neurodegeneration.

